# Relationship Between Child Perfectionism and Psychological Disorders

**DOI:** 10.3389/fpsyg.2019.01855

**Published:** 2019-09-06

**Authors:** Luis Manuel Lozano, Inmaculada Valor-Segura, Eduardo García-Cueto, Ignacio Pedrosa, Alexia Llanos, Luis Lozano

**Affiliations:** ^1^Department of Methodology of Behavioral Sciences, University of Granada, Granada, Spain; ^2^Mind, Brain and Behavioral Research Center, University of Granada, Granada, Spain; ^3^Department of Social Psychology, University of Granada, Granada, Spain; ^4^Department of Psychology, University of Oviedo, Oviedo, Spain; ^5^Independent Researcher, Oviedo, Spain

**Keywords:** perfectionism, clinical symptomatology, childhood, mediation analyses, childhood perfectionism inventory

## Abstract

**Objective:** Perfectionism is one of the variables related to the correct emotional development or with the appearance of clinical symptomatology in childhood. A study has been designed to evaluate the differential effect that each dimension of perfectionism (external pressure, self-exigency, and negative self-evaluation) has in a Spanish children sample of general population for each of the following clinical aspects: irritability, worthlessness feelings, thinking problems, and psychophysiological symptoms.

**Method:** By a random cluster sampling, a total of 2,636 children from 8 to 12 years (*M* = 9.9, SD = 1.2; 51.3% boys) took part in this research. A serial multiple mediators model was used to check the relation between external pressure over the clinical symptoms through self-exigency and negative-self-evaluation.

**Results:** The results have shown a predictive effect of external pressure over a great variety of clinical symptomatology (irritability, worthlessness, thinking problems, and psychophysiological symptoms), a relation mediated by self-exigency and negative self-evaluation. These relations suggest that external pressure and negative self-evaluation are maladaptive dimensions as they predict the appearance of symptomatology, being the level of self-exigency a protective dimension and favoring the child’s positive development.

**Conclusions:** In consequence, these results point to the importance of the study of these variables that can generate difficulties in childhood in order to improve children’s quality of life and their correct development.

Perfectionism is a construct that has received growing attention during the last decade (Ayearst et al., 2012; Lloyd et al., 2014; Sherry et al., 2014; Hong et al., 2016; Gäde et al., 2017; Schmidt et al., 2018; Bouguettaya et al., 2019; Curran and Hill, 2019). It is defined as a disposition of the personality that is characterized by the search of faultlessness and the establishment of very high levels of performance, together with excessively critical self-evaluations (Frost et al., 1990; Hewitt and Flett, 1991). The perfectionist person self-assesses herself or himself not only through the achievement or success that is obtained after the implementation of the task but also, and as a very relevant factor, through others’ acceptance and approval (Flett and Hewitt, 2002; DiBartolo and Varner, 2012). Intrapersonal as well as interpersonal aspects are fundamental in order to understand perfectionism in its full extent (Hewitt et al., 2003).

With the aim to evaluate this construct, Frost et al. (1990) developed the Multidimensional Perfectionism Scale (FMPS) that includes the following dimensions: concern over mistakes, personal standards, parental expectations, parental criticism, and doubts about actions and organization; even though a high level of personal standards is considered to be the central element of perfectionism. Besides, Hewitt and Flett (1991) developed the Multidimensional Perfectionism Scale (HMPS) assuming three dimensions: self-oriented perfectionism, perfectionism oriented to others, and socially prescribed perfectionism.

The variety of dimensions that the instruments show is more apparent than real (Enns and Cox, 2002). Empirically, the different dimensions that have been proposed are supported by two great underlying dimensions: a positive dimension (or perfectionistic strivings), formed by the levels of personal standards, organization, self-oriented perfectionism, and perfectionism oriented to others; and a negative dimension (or perfectionistic concern) formed by concern over mistakes, doubts about actions, parental criticism, parental expectations, and socially prescribed perfectionism (Frost et al., 1993; Stöeber and Otto, 2006).

The majority of research into how the dimensions of perfectionism affect the psychological field has used adolescent and adult samples (Essau et al., 2008; Flett et al., 2011; Smith et al., 2014). This, produce a lack in the research about children, either about how the perfectionism dimensions influence the emotional disorders (e.g., Hewitt et al., 2002; Rice et al., 2007; Flett and Hewitt, 2012), or in the cognitive variables (DiBartolo and Varner, 2012).

From the multidimensional conception that this study assumes (Lozano et al., 2012), it is considered that the positive dimension of child perfectionism is formed by Self-Exigency (SE), and the negative one by External Pressure (EP), and Negative Self Evaluation (NSE). According to Bandura’s theory (1986), perfectionist traits will be developed through interactions between a child’s characteristics and his/her social environment (primarily family and school). This environment exerts perfectionist pressures (EP in the model) *via* social expectations of perfection and criticism when these expectations are not met. In these conditions, children put high standards of excellence on themselves (SE, according to the model) due to adults’ behavioral models and the selective reinforcement that these models exert when children achieve levels of excellence (Cole et al., 2001). When there are differences between a child’s task implementation level and his/her standards (Choy and McInerney, 2006) or when the family or school environment is judgmental, unpredictable or hostile (Herman and Ostrander, 2007), the child may develop NSE. Thus, perfectionism pressures of the external environment favor the appearance of other aspects of perfectionism (Morris and Lomax, 2014).

Studies about how the different dimensions of perfectionism affect the psychological balance of children show that higher sensitivity to making mistakes is associated with a decline in the levels of happiness and satisfaction and with a higher emotional instability (Rice and Preusser, 2002; Loades et al., 2019). Self-oriented perfectionism (SE in this study) and socially prescribed perfectionism (EP in this study) are also associated with high levels of anxiety and depression (Hewitt et al., 2002; Stornelli et al., 2009). However, other studies point that EP is positive and significantly associated with high levels of anxiety and depression, but SE is protective against emotional disorders when there is acceptance of the error (Lozano et al., 2015).

Due to the lack of studies about the effect that perfectionism has on child emotional aspects, this study attempts to analyze the relationship between perfectionism and child symptomatology that may hinder a balanced development, such as irritability, feelings of worthlessness, thinking problems, and psychophysiological symptoms. Thus, in line with adolescent and adult population situation (Stöeber and Otto, 2006), it is hypothesized that an increase in the dimensions that shape the negative field of child perfectionism (EP and NSE) will, therefore, favor an increase in the symptomatology of irritability, feelings of worthlessness, thinking problems, and psychophysiological symptoms. In contrast, it is expected that the positive field of perfectionism (SE) will be a protective element against the symptomatology that is studied.

## Materials and Methods

### Participants

A random cluster sample by the primary education schools of the Principality of Asturias (Spain) was performed. The sample consisted of 2,636 children between the ages of 8 and 12 years (*M* = 9.9, SD = 1.2). 51.3% were boys; 22.8% were in third grade, 25.4% were in fourth, 26.2% were in fifth, and 25.6% in sixth grade.

The cases where missing values were observed in the answers to the different questionnaires have been deleted (Fernández-Alonso et al., 2012). Table 1 shows the descriptives of the sample for the study of the different clinical variables in general and by grade, together with the maximum error made in the estimations.

**Table 1:** Descriptive statistics of the clinical variables and maximum error of estimate.

Clinical variables		Age		% Grade				ME
	*n*	*M*	SD	3rd	4th	5th	6th	
Worthlessness	2,507	9.93	1.23	22.2	25.1	26.5	26.2	±1.88
Irritability	2,513	9.91	1.22	22.3	25.4	26.3	25.9	±1.88
Thinking problems	2,550	9.89	1.22	22.8	25.4	26.4	25.5	±1.86
Psychophysiological symptoms	2,413	9.91	1.23	22.4	25.1	26.4	26.2	±1.91

*n = sample size; *M* = mean; SD = standard deviation; ME = maximum error of estimate (CL = 95%)*.

### Instruments

The following assessment instruments have been applied:

The Childhood Perfectionism Inventory (IPI, in Spanish; Lozano et al., 2012). This questionnaire of 25 items evaluates the following dimensions: external pressure (EP; *α* = 0.90): The child perceives that her or his closest environment demands perfect behavior of her or him (e.g., “I must do things better than anyone else for others to value me”). Self-exigency (SE; *α* = 0.82): assesses the perfectionist attitude with which the child faces tasks (e.g., “I try to be the best in everything I do”). Negative self-evaluation (NSE; *α* = 0.90): evaluates the presence of negative self-judgments when the execution is not as excellent as it is wanted (e.g., “When I do not do things as well as I want, I feel like I am good for nothing”).

The Educational-Clinical Questionnaire (CECAD, in Spanish; Lozano et al., 2011). It assesses the following clinical aspects: worthlessness (*α* = 0.91): The perception that every child has of his or her worth and capacity to face daily tasks (e.g., “I think I am good for nothing”). Irritability (*α* = 0.87): evaluates the perceived capacity to get angry and the inner feeling of anger in everyday situations (e.g., “Anything irritates me very much”). Thinking problems (*α* = 0.83): It is valued if the person feels inundated by intrusive thoughts; if there is a tendency to value the things from the most negative perspective and if there is fear to lose control over the things that may happen (e.g., “I tend to think the worst”). Psychophysiological symptoms (*α* = 0.88): assess the level of physiological activation that accompanies anxiety: breathing problems, difficulties to maintain sleep, shaky hands, and palpitations (e.g., “I feel a pressure in my chest that leaves me out of breath”).

All the questionnaires have a format of 5-point Likert scale as this is the number that maximizes their psychometric properties (Lozano et al., 2008). These questionnaires have no reversed items in order to avoid any possible bias due to the reading skills of the sample (Suárez-Álvarez et al., 2018). In all dimensions, a high score is associated with a greater level in the variable.

### Procedure

The different questionnaires were applied by two psychologists who are experts in the use of questionnaires with children. The questionnaires were provided in a single booklet, which was given to children in the classroom where they attend class without ever exceeding 1 h. In a complementary way, data about sociodemographic aspects were also collected. In all the cases, the questionnaires were filled anonymously, and participation in the study was entirely voluntary.

Before administering the questionnaires, consent from all the children’s parents was requested to allow the children to participate in the research.

### Data Analysis

**Figure 1:**
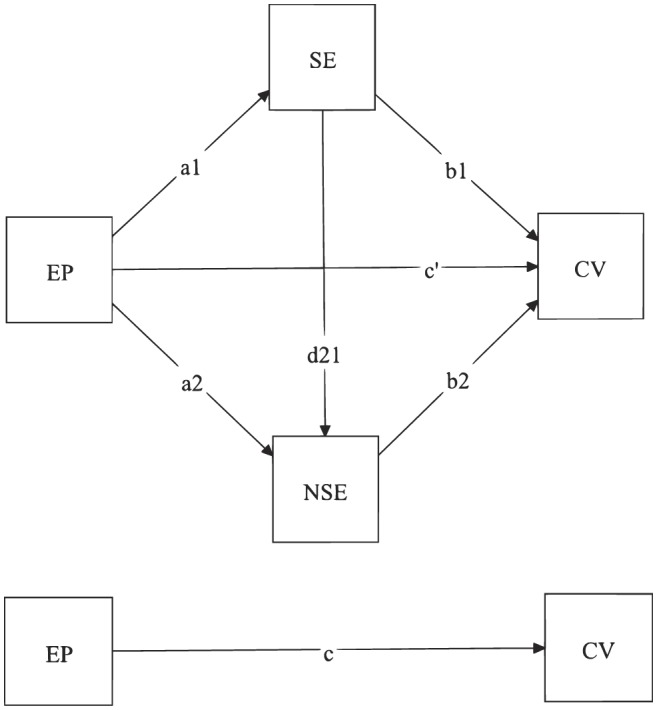
Illustration of the proposed model with two serial mediators. Note: EP, External Pressure; SE, Self Exigency; NSE, Negative Self-Evaluation; CV, Clinical Variables (Worthlessness, Irritability, Thinking problems, and Psychophysiological symptoms).

To test the aforementioned hypotheses, a serial multiple mediators model was used, as represented in Figure 1.

These models attempt to predict the symptomatology score of the clinical aspects that are studied by the assessment of one direct (*c*′) and three indirect effects (I1: EP → SE → Clinical Variables [*a*_1_*b*_1_]; I2: EP → SE → NSE → Clinical Variables [*a*_1_*d*_21_*b*_2_]; I3: EP → NSE → Clinical Variables [*a*_2_*b*_2_]). The sum of the direct effect with the three indirect effects is the total effect, which is represented as “*c*” in Figure 1.

Given that the proposed model assumes a linear relation between two mediators, the partial correlation between both mediators was calculated while controlling the effect of EP. Therefore, if this correlation were significant (CL = 95%), the use of this model would be justified, as both mediators were related even after adjusting for the effect of EP (Hayes, 2013).

Using the PROCESS software (Hayes, 2013) implemented on SPSS 20.0, 95% bootstrap bias-corrected confidence intervals (BCI) were generated for the direct and conditional effects on the basis of 10,000 bootstrap samples. Effects are statistically significant when 0 is not included in the bootstrap interval. Following Cumming’s recommendations (Cumming, 2014), intervals for all the estimated parameters are reported.

## Results

First, the partial correlation between the mediators was calculated while controlling for the effect of EP. The value that was obtained was *r*_SE.NSE.EP_ = 0.292, 95% CI [0.257, −0.327], *p* < 0.001. Given that the partial correlation differed from 0, the use of a model with serial mediators is justified.

In Tables 2–5 below, the values of the coefficient of regression are shown together with the total and indirect effects that EP has on the different clinical variables that are studied (worthlessness, irritability, thinking problems, and psychophysiological symptoms).

**Table 2:** Estimates of worthlessness mediation.

*R*^2^ = 0.392 *F*_32,503_ = 319.99 (*p* < 0.001)				
	Coefficient	Standard error	95% Confidence interval	
*a*_1_	0.494^**^	0.021	0.454	0.535
*a*_2_	0.487^**^	0.025	0.438	0.535
*d*_21_	0.293^**^	0.020	0.255	0.331
*b*_1_	−0.129^**^	0.016	−0.160	−0.099
*b*_2_	0.475^**^	0.020	0.437	0.513
*c*′	0.149^**^	0.022	0.106	0.193
*c*	0.385^**^	0.021	0.343	0.427
**Indirect effects**	**Effect**	**95% Confidence interval**		
Total	0.236	0.203	0.269	
I1	−0.064	−0.081	−0.049	
I2	0.069	0.058	0.081	
I3	0.231	0.202	0.261	

*Regression coefficients make reference to those shown in Figure 1*.

*I1: EP → SE → Clinical Variable*.

*I2: EP → SE → NSE → Clinical Variable*.

I3: EP → NSE → Clinical Variable.

** *p < 0.001*.

**Table 3:** Estimates of irritability mediation.

*R*^2^ = 0.277 *F*_32,509_ = 2105.33 (*p* < 0.001)				
	Coefficient	Standard error	95% Confidence interval	
*a*_1_	0.496^**^	0.021	0.455	0.536
*a*_2_	0.506^**^	0.025	0.457	0.554
*d*_21_	0.294^**^	0.020	0.255	0.333
*b*_1_	−0.015	0.014	−0.042	0.011
*b*_2_	0.273^**^	0.016	0.242	0.304
*c*′	0.121^**^	0.018	0.085	0.158
*c*	0.291^**^	0.016	0.260	0.324
**Indirect effects**	**Effect**	**95% Confidence interval**		
Total	0.170	0.146	0.197	
I1	−0.008	−0.021	0.006	
I2	0.040	0.033	0.048	
I3	0.138	0.118	0.159	

*Regression coefficients make reference to those shown in Figure 1*.

*I1: EP → SE → Clinical Variable*.

*I2: EP → SE→ NSE → Clinical Variable*.

*I3: EP → NSE → Clinical Variable*.

** *p < 0.001*.

**Table 4:** Estimates of thinking problems mediation.

*R^2^* = 0.375 *F*_32,546_ = 409.91 (*p* < 0.001)				
	Coefficient	Standard error	95% Confidence interval	
*a*_1_	0.491^**^	0.020	0.451	0.531
*a*_2_	0.503^**^	0.025	0.454	0.551
*d*_21_	0.296^**^	0.020	0.257	0.334
*b*_1_	−0.024	0.015	−0.054	0.006
*b*_2_	0.407^**^	0.016	0.375	0.438
*c*′	0.13^**^	0.019	0.092	0.169
*c*	0.382^**^	0.018	0.347	0.418
**Indirect effects**	**Effect**	**95% Confidence interval**		
Total	0.252	0.223	0.282	
I1	−0.012	−0.026	0.002	
I2	0.059	0.050	0.070	
I3	0.205	0.180	0.231	

*Regression coefficients make reference to those shown in Figure 1*.

*I1: EP → SE → Clinical Variable*.

*I2:EP → SE → NSE → Clinical Variable*.

*I3 EP → NSE → Clinical Variable*.

** *p < 0.001*.

**Table 5:** Estimates for psychophysiological symptoms mediation.

*R*^2^ = 0.179 *F*_32,409_ = 237.34 (*p* < 0.001)				
	Coefficient	Standard error	95% Confidence interval	
*a*_1_	0.494^**^	0.021	0.452	0.535
*a*_2_	0.502^**^	0.025	0.452	0.552
*d*_21_	0.296^**^	0.020	0.257	0.335
*b*_1_	−0.099^**^	0.030	−0.157	−0.041
*b*_2_	0.556^**^	0.031	0.495	0.617
*c*′	0.378^**^	0.038	0.305	0.452
*c*	0.690^**^	0.034	0.622	0.757
**Indirect effects**	**Effect**	**95% Confidence interval**		
Total	0.312	0.263	0.362	
I1	−0.049	−0.077	−0.021	
I2	0.081	0.067	0.098	
I3	0.279	0.241	0.321	

*Regression coefficients make reference to those shown in Figure 1*.

*I1: EP → SE → Clinical Variable*.

*I2: EP → SE → NSE → Clinical Variable*.

*I3: EP → NSE → Clinical Variable*.

** *p < 0.001*.

As can be seen in the tables (Tables 2–5), all the coefficients (from *a*_1_ to *b*_2_) are statistically significant, so there is a relation between the predicting variable and what it predicts, except for the relation of SE with irritability and thinking problems (coefficient *b*_1_ in Tables 3, 4, respectively).

When examining the effect of each unhealthy perfectionism variable over each of the clinical aspects, it was observed that the relations of the EP as well as of the NSE favor the appearance of the symptoms since the value of the coefficients is positive. In the same line, it can be observed that the total effect of EP over each of the clinical variables is significantly positive. This value consists of the direct effect of EP (*c*′) as well as the sum of all the indirect effects in the model.

As almost all the indirect effects are statistically significant, these results represent evidence in favor of the mediation effect of SE and NSE over the effect of EP over the clinical variables.

It is also observed that the indirect effects EP → SE → NSE → Clinical Variable and EP → NSE → Clinical Variable are positive, which means that they provoke an increase in the clinical symptomatology.

Similarly, in relation with the second hypothesis, it is observed that the effect of SE over worthlessness (*b*_1_ = −0.129; 95% CI [−0.160, −0.099]) and psychophysiological symptoms (*b*_1_ = −0.099; 95% CI [−0.157, −0.041]) are negative, the same as the indirect effect EP → SE → Clinical Variable. The different coefficients show that the higher the EP, the higher SE is (given that *a*_1_ is positive), and this increase in SE is associated with a decrease (given that *b*_1_ is negative) of the clinical symptomatology which points to the fact that it behaves as a protective dimension against these symptomatologies. When we compare the size of the indirect effects by pairs (see Table 6), it is observed that for each of the clinical variables, the biggest of the indirect effects is that generated by EP → NSE → Clinical Variable, followed by EP → SE → NSE → Clinical Variable. The smallest protective effect generated by EP being the smallest the protective effect generated by EP → SE → Clinical Variable.

**Table 6:** Comparison of the magnitude of indirect effects of SE and negative self-evaluation.

	Worthlessness			Irritability		
	Effect	95% Confidential interval		Effect	95% Confidential interval	
I1-I2	−0.133	−0.156	−0.112	−0.047	−0.064	−0.032
I1-I3	−0.295	−0.332	−0.262	−0.146	−0.172	−0.120
I2-I3	−0.163	−0.193	−0.132	−0.098	−0.120	−0.079
	**Thinking problems**			**Psychophysiological symptoms**		
	**Effect**	**95% Confidential interval**		**Effect**	**95% Confidential interval**	
I1-I2	−0.071	−0.090	−0.053	−0.130	−0.166	−0.095
I1-I3	−0.216	−0.248	−0.186	−0.328	−0.380	−0.277
I2-I3	−0.146	−0.173	−0.119	−0.198	−0.238	−0.160

*I1: EP → SE → Clinical Variable*.

*I2: EP → SE → NSE → Clinical Variable*.

*I3: EP → NSE → Clinical Variable*.

## Discussion and Conclusions

This study attempts to assess, in a general population sample with ages from 8 to 12 years, the relation that exists between child perfectionism dimensions and the intrapersonal problems of Worthlessness, Irritability, Thinking problems and Psychophysiological symptoms.

As a global conclusion, it has been shown that perfectionism is related to the occurrence of the psychological problems studied similarly to what it does with anxiety and child depression (Hewitt et al., 2002; McCreary et al., 2004; Stornelli et al., 2009; Lozano et al., 2015).

The results reaffirm the first hypothesis that EP and NSE are unhealthy perfectionism dimensions since they are significantly associated with the increase of the clinical symptomatology. Moreover, EP favors the development of an NSE (ideas of inferiority when compared with their peers, doubts if they have performed well in the tasks, as well as the non-acceptance of errors). It is also relevant to observe that the most important effect over the internalized problems that are studied is the one exerted by the interaction of the two dimensions that shape unhealthy perfectionism (EP and NSE).

These results are consistent with social learning models (Bandura, 1986) as well as with the cognitive theory of Beck et al. (1979). First, children learn in their relationship with their parents and teachers that, in order to be valued, they must meet the high expectations of achievement that are demanded from them. Therefore, their effort is permanently directed to reach that level of excellence in order to be approved and accepted. In this way, a cognitive pattern is being formed—perfectionism—that children use with regularity to judge everything that is related to the tasks they perform. When they make mistakes that are not accepted in their environment, this cognitive scheme is activated, favoring distorted judgments of reality that, in turn, triggers the occurrence of emotional suffering. Among the cognitive distortions that may appear when performing a task that is well but not perfectly executed, it is possible to find dichotomous thinking (“the task is not perfectly done then I am good for nothing”), magnification (“I have made a mistake, they are not going to love me”), “should” enunciations (“I should do it better”), incorrect labeling (“I have made a mistake, I am a loser”), etc. Therefore, the perfectionism scheme in children turns to be an element of cognitive vulnerability. According to the *diathesis-stress model* by Clark and Beck (2010), this vulnerability could emerge in situations in which children’s vital interests of *approval, acceptance, independence, and competence* are at potential risk, thus favoring the occurrence of the internalized symptomatology that is studied (e.g., Rice et al., 2015). If children feel incompetent to handle any demanding situation (diathesis), it can be interpreted as dangerous (stress), thus leading to self-perceptions of worthlessness and incompetence (i.e., I think I do everything wrong), irritability (i.e., I feel anger inside) and therefore increasing the thinking problems (i.e., “disturbing thoughts come to my mind, even if I don’t want”) as well as the corresponding physiological symptoms (e.g., “I feel a pressure in my chest that leaves me out of breath”).

Consistently with what Stöeber and Otto (2006) argue about perfectionist strivings, it is confirmed that SE is a protective variable that has a positive direct effect (when it increases, the symptomatology of worthlessness and psychophysical symptoms decrease) as well as an indirect effect (when it has a mediating effect between EP and NSE, it reduces the negative effect that this interaction causes in all the variables that are studied). A child with high SE that is presented with a task she or he feels capable of doing well without fear of making mistakes, with the certainty that she or he is going to be accepted and loved even in the case of failing at first, does not feel vulnerable when facing such a challenge, as there is no threat for the child’s approval and acceptance, and ultimately, the studied psychological problems do not appear.

In conclusion, this study does not support the theoretical position where perfectionism is always regarded as maladaptive and an indicator of a psychological maladjustment (Flett and Hewitt, 2006), since it can also be adaptive and healthy as it has also been shown in previous studies (Stöeber and Otto, 2006; Owens and Slade, 2008; Lozano et al., 2015).

These results have multiple practical consequences not only from a clinical perspective but also educative. Both approaches have to direct their efforts to develop a high intrapersonal intelligence, resilient children, able to face problems and not avoid or escape from them due to fear of failure. It is necessary to plan cooperative, nurturing, and not exclusively competitive contexts where adults express in a direct way their positive expectations of the child’s capacity and achievements (Kenney-Benson and Pomerantz, 2005; Hutchinson and Yates, 2008), together with a non-judgmental attitude toward errors, in order to favor the development of high self-esteem (McArdle and Duda, 2004) and to avoid worries or negative beliefs about themselves which can develop anxiety disorders in adults (Esbjørn et al., 2015).

This study also has limitations that could be transformed into future research directions. It would be relevant to supplement children’s perfectionism self-reports with teachers’ and parents’ opinions in order to examine if parent’s attributions could predict child perfectionism problems as have been found in relation with others disorders (e.g., Williamson and Johnston, 2015) and to use clinical and not only general population to generalize these results in child population too. Just as there is a large number of retrospective studies whose aim is to determine how parenting styles affect the development of perfectionism and emotional disorders (e.g., Yoon and Lau, 2008; Speirs Neumeister et al., 2009), it would be important to conduct them longitudinally as well, with the aim of determining how child perfectionism may affect the development of emotional disorders in adulthood.

## Data Availability

The datasets generated for this study are available on request to the corresponding author.

## Ethics Statement

This study was carried out in accordance with the recommendations of University of Granada, ethic committee with written informed consent from all subjects. All subjects gave written informed consent in accordance with the Declaration of Helsinki. The protocol was approved by the University of Granada, ethic committee.

## Author Contributions

LL and AL contributed conception and design of the study. IP and EG-C organized the database. LL and IV-S performed the statistical analysis and wrote the first draft of the manuscript. All authors contributed to manuscript revision, read and approved the submitted version.

### Conflict of Interest Statement

The authors declare that the research was conducted in the absence of any commercial or financial relationships that could be construed as a potential conflict of interest.
